# Traumatic Shock and Hemorrhage

**Published:** 1918-12

**Authors:** 


### TRAUMATIC SHOCK AND HEMORRHAGE

The Medical Research Laboratory has never been used to the same extent in any previous war as in the world conflict that has just ended; and as a result of the correlation and co-ordination of the work of laboratory and research men with that of clinicians in the army, scientific medicine and surgery have made many wonderful advances. The Chief Surgeon and the Director of the Laboratory Division of the American Expeditionary Forces, have had the vision to realize their opportunities for applying research investigations to practical medical and surgical problems; and, as a result of the establishment of the Division of Surgical Research in the Central Medical Department Laboratory, surgical technic has developed and improved in a way that has been of great practical benefit to the surgeons who have treated our wounded soldiers, and which may be applied in the treatment of surgical cases in civil life.

The Division of Laboratories and Infectious Diseases, since the beginning of our participation in the war, has been making scientific investigations regarding a number of phases of war wounds, and the reports of their researches as applied to the treatment of various conditions have been valuable contributions to the medical literature of the war. *War Medicine* is privileged to publish in this number a practical treatise on "Traumatic Shock and Hemorrhage" that has been prepared by the Division of Surgical Research of the Central Medical Department Laboratory of the American Expeditionary Forces. This article has very few of the ear marks of the research laboratory, but is altogether a very practical discussion on perhaps the most important phase of war wounds. For instance, keeping the wounded man warm is a very practical and important part of the treatment of shock. It is discussed in this treatise in a way that would impress any layman, as well as the most scientific surgeon, with the simplicity of the measures that have been found to give the best results. Every person who touches the wounded man, from the stretcher bearer or private who applies first aid dressing to the surgeon who performs the operation, should have drilled into him, until it cannot be forgotten in any emergency, the necessity for keeping wounded soldiers warm.

### Blood Pressure in Shock.

The critical level of a falling blood pressure and its modification by hemorrhage, with its relation to the alkali reserve and the oxygen supply of the blood, is described so simply in this treatise on shock and hemorrhage that the indications for the treatment, which are outlined, are made very clear. The methods of blood transfusion and the selection of donors are also discussed in a way that make them seem very easy of application. The technic of preparing and administering gum salt solution is also described; but judging from the experiences of a number of surgeons who, at the November meeting of the Research Society, reported deaths following its use, gum salt transfusion should not be employed, except perhaps in selected cases during the early hours after a wound or injury. Surgery in its relation to shock is another subject that is dealt with, which will be helpful to those who have the responsibility of treating wounded men.

The Central Medical Department Laboratory has prepared transfusion outfits by the hundreds, and every evacuation and advanced hospital for the seriously wounded is supplied with all the apparatus and equipment needed for this life saving procedure. The reagents necessary for testing donors and sterilized needles and cannulae are ready for instant use. A resuscitation officer, whose duties are outlined in this paper, and who has been especially trained for treating shock, has been assigned to each unit.

The methods of treating shock and hemorrhage, which medical officers have learned during their military service, and which are an improvement over those in use prior to the war, will continue to be used by American surgeons in their private practice when they return to take up their home work.

The practical benefits of the research laboratory have been proved by the investigations that have been carried out on ascale in this colossal conflict that was never dreamed of before, and the improvements in medical and surgical technic that have resulted from them are among the compensations that will come from even so dreadful a thing as war.

The Division of Surgical Research in the Central Medical Department Laboratories, in simplifying and standardizing the methods for treating shock and hemorrhage, has performed a service of very great value to the American Army. Many lives of soldiers, who have proved themselves to be the best and the bravest of patriots, have been saved, that would have been lost had not this research work been done.

